# Neuromuscular behaviour in the first dorsal interosseus following mental fatigue

**DOI:** 10.1113/EP091349

**Published:** 2023-12-21

**Authors:** Michael J. Marsala, Anita D. Christie

**Affiliations:** ^1^ School of Kinesiology, Faculty of Health Sciences The University of Western Ontario Ontario Canada

**Keywords:** EMG, mental fatigue, motor unit firing rate, sex differences, sustained attention

## Abstract

We examined sex‐specific changes to neuromuscular function in response to mental fatigue. Twenty‐five young, healthy adults (13 F, 12 M) performed a mentally fatiguing task and control condition for 30 min on two separate days. Neuromuscular function was assessed in the first dorsal interosseous before and after each condition. Reaction time decreased after the mentally fatiguing task (*P* < 0.001, η^2^ = 0.47). Males and females reported higher levels of subjective fatigue after the mentally fatiguing task (*P* < 0.02, η^2^ = 0.07). Motor unit firing rate increased over time at 10% maximal voluntary contraction (MVC; *P* < 0.04, η^2^ = 0.16), and decreased over time at 50% MVC (*P* < 0.01, η^2^ = 0.14); however, this was not unique to either sex. During a variable force contraction, error decreased in females over time and increased in males (*P* < 0.05, η^2^ = 0.13), although changes were not unique to mental fatigue. Physiological function of the neuromuscular system was not specifically affected by mental fatigue in males or females.

## Abstract (highlights)

- **What is the central question of this study?**
  What is the effect of mental fatigue on neuromuscular behaviour in the first dorsal interosseus?
- **What is the main finding and its importance?**
  Mental fatigue does not have a significant effect on neuromuscular function in the first dorsal interosseus. This evidence supports previous evidence observed in the tibialis anterior that there is no significant influence on, nor sex‐specific changes to, neuromuscular function after a mentally fatiguing condition.

## 1 INTRODUCTION

Mental fatigue is a psychophysiological state that occurs after prolonged periods of cognitive activity or sustained attention (Boksem et al., 2005). It can lead to an inability to efficiently pay attention to a task (Boksem & Tops, 2008; Boksem et al., 2005) and is characterized by a combination of subjective ratings of increased fatigue and perceived exertion, or behaviourally through changes to attention to a task (Boksem & Tops, 2008; Boksem et al., 2005; Lim et al., 2010; van Cutsem et al., 2017) (Boksem et al., 2005; Van Cutsem et al., 2017). Boksem and colleagues (2008) described the process of mental fatigue as a subconscious feeling when the energy cost of a task outweighs the perceived benefits. These changes in perception of effort reduce motivation and alertness (Boksem et al., 2005; Lorist et al., 2002; van der Linden & Eling, 2006) and result in a reduction in attention that can lead to increased reaction time (Marcora et al., 2009) and greater task error (Lorist et al., 2000).

Further assessments of mental fatigue have demonstrated reductions in endurance performance (Pageaux & Lepers, 2018; van Cutsem et al., 2017), sport‐related decision making and psychomotor performance (Duncan et al., 2015; Habay et al., 2021). For example, a reduction in time to exhaustion was observed in participants who had an increased perception of effort in a cycling task after 30–90 min of a mentally fatiguing task, despite no changes in cardiorespiratory mechanisms or blood lactate measurements (Marcora et al., 2009; Pageaux et al., 2015). In another study on self‐paced endurance, running speed was reduced and participants reported higher levels of perceived exertion during the exercise task after 30 min of an incongruent Stroop task with no change in heart rate or blood lactate (Pageaux et al., 2013). The lack of observed changes in cardiorespiratory factors amidst performance reductions suggest other factors contribute to the changes in performance with mental fatigue.

Previous research on neuromuscular properties has shown that mental fatigue may influence performance of submaximal, but not maximal, isometric contractions, but not contractile properties (Kowalski & Christie, 2020; Kowalski et al., 2022; Pageaux et al., 2015). In submaximal isometric handgrip tasks, increased surface electromyographic (EMG) activity and a reduced time to exhaustion were observed, with no change in EMG amplitude observed during a maximal effort (Bray et al., 2012, 2008). In contrast, a reduction in surface EMG amplitude was observed in the tibialis anterior at 10% maximal voluntary contraction (MVC), with no change in motor unit firing rate after a mentally fatiguing task (Kowalski et al., 2022). Collectively, these results indicate that the consequences of mental fatigue for neuromuscular function remain unclear and are potentially different between muscles.

Outcomes of neuromuscular function can vary across muscles, and physiological differences both in the spinal cord and at the muscular level can influence motor unit firing rates. The difference in muscles due to motoneuron size distributions and muscle fibre type affect the proportion of recruitment and firing rate contributions to specific force output for each muscle (del Vecchio et al., 2018; Moritz et al., 2005). The first dorsal interosseous (FDI) and the tibialis anterior (TA), specifically, have different ranges of MVC at which recruitment of new motor units saturates. In the FDI, recruitment has been reported to occur up to 70% MVC (De Luca & Hostage, 2010) and in the TA up to 90% MVC (van Cutsem et al., 1997). The differences in recruitment ranges can change the contribution of firing rate to force output (De Luca et al., 1982; Feiereisen et al., 1997; Milner‐Brown et al., 1973). Relative to the FDI, the TA has a higher innervation ratio, greater cross‐sectional area and a different primary function (fine motor control vs. gait) (Allen et al., 2014; Duchateau & Enoka, 2022; Johnson et al., 1973).

In addition to structural and functional differences between muscles, differences in input from the motor cortex may also exist. Common synaptic input to motor neuron pools changes in response to a cognitive load (Pereira et al., 2019) and varies across muscles (Farina & Negro, 2015) impacting force and motor unit firing rate variability (Pereira et al., 2019). Such differences in the input to motor neuron pools, combined with the anatomical, physiological and functional differences between the FDI and TA, suggest the neuromuscular response to a mentally fatiguing task may differ across these muscles.

To date, motor unit firing behaviour in response to mental fatigue has only been assessed in the TA. Other studies including surface EMG measures during mental fatigue in the upper limb have been performed on the forearm during handgrip tasks (Bray et al., 2012, 2008). These studies have employed static isometric contractions at an assigned force level. These static contractions provide clear measurements for neuromuscular function but lack applicability to the variable motion of daily activities or exercise. Motor unit firing behaviour is different during various phases of a contraction depending on the trajectory of the force output (Knight & Kamen, 2004; Park et al., 2016). Variable force contractions and the associated modulation of firing rates could therefore have different responses from static contractions to mentally fatiguing conditions.

Sex‐related differences in neuromuscular behaviour and the perception of fatigue may also cause varying responses to mental fatigue (Pageaux & Lepers, 2018). Males and females differ in force output, with more variable force (Jakobi et al., 2018) and greater increases in variability during a cognitive task (Pereira et al., 2019) in females compared with males. Motor unit firing rates are also different between sexes, with females having higher and more variable motor unit firing rates at similar force levels (Inglis & Gabriel, 2021; Lulic‐Kuryllo & Inglis, 2022). Females also tend to report higher subjective ratings of fatigue (Engberg et al., 2017). Higher ratings of subjective fatigue and perceived exertion as well as differences in neuromuscular function and responses to cognitive tasks suggest that there could exist sex‐specific changes in response to a mentally fatiguing task. In a previous study, a sex‐specific response was observed, in which motor unit firing rates declined following mental fatigue in males but not females, during contractions at 50% MVC (Kowalski & Christie, 2020). However, a follow‐up study found no sex differences (Kowalski et al., 2022).

The purpose of this study was to further explore sex‐related differences in the impact of mental fatigue on neuromuscular function in the first dorsal interosseous (FDI) during both static and variable force contractions. We sought to expand on previous assessments using a variable force contraction in the FDI. As task error increases with mental fatigue (Lorist et al., 2000) we hypothesized that the performance of a complex force tracing task would show an increase in error after a mentally fatiguing task. As well, we hypothesized that females would have a larger increase in task‐error in conjunction with their higher force variability, and greater increases in motor unit firing variability in the presence of mental fatigue, compared to males.

## 2 METHODS

### 2.1 Ethical approval

Prior to enrolment, each participant provided written informed consent according to procedures approved by the Health Sciences Research Ethics Board at the University of Western Ontario and the study conformed to the standards set by the *Declaration of Helsinki*.

### 2.2 Participants

Twenty‐five participants, aged 23 ± 2.9 years, were recruited from the local university community (13 female and 12 male). All participants reported normal or corrected‐to‐normal vision, were free of any musculoskeletal or neurological impairments, and were not taking any medication which may alter cognitive or neuromuscular function.

### 2.3 Experimental protocol

Prior to starting the protocol, all participants completed three surveys, the Pittsburgh Sleep Quality Index (PSQI), the Multidimensional Fatigue Index (MFI) and the International Physical Activity Questionnaire (IPAQ). Participants then visited the lab on two separate days, one day where the protocol was performed with a mentally fatiguing task, and another day for a control condition. The order of conditions was randomized across days.

On the first visit to the lab, participants were acquainted with the set‐up and after obtaining an initial MVC a practice session was performed. The practice session consisted of 12 force tracing tasks. The first and last trial was a series of contractions to plateaus at 10%, 20% and 50% MVC for 1 s each, and the middle 10 trials were a sine wave force template, centred on 20% MVC. This practice session was performed on the first visit only. The experimental protocol consisted of participants tracing a series of steady force and variable force (sine wave) templates before and after a mentally fatiguing task or control condition. The order of the conditions and force tracings were randomized for each participant. An MVC was then performed at the end to assess fatigue.

Self‐reported fatigue on a 10‐point Likert scale was recorded (1 = not tired at all, 10 = very tired; Kowalski & Christie, 2020) before and after the mentally fatiguing and control conditions.

### 2.4 Questionnaires

The PSQI is a self‐report questionnaire used to measure sleep and sleep quality in seven domains: subjective sleep quality, sleep latency, sleep duration, habitual sleep efficiency, sleep disturbances, sleep medication use, and daytime dysfunction. For each question, participants provide a rating from 0 to 3 with lower scores indicating healthier sleep and a total score across all questions of 5 or above indicates poor sleep quality (Buysse et al., 1989). The MFI is used to assess general fatigue in a self‐report questionnaire of 20 items, with higher scores indicating greater fatigue (Smets et al., 1995). The IPAQ is used to gather data on health‐related physical activity in five domains: job‐related physical activity, transportation physical activity, housework/house maintenance and family physical activity, recreational/sport/and leisure‐time physical activity, and time spent sitting.

### 2.5 Sustained attention task

Participants performed the Psychomotor Vigilance Task (PVT) to induce mental fatigue. Participants were seated 2 m away from a computer monitor used to display the PVT. Every 2–10 s, a red number counter on a black screen appeared and began to count up in milliseconds (Dinges & Powell, 1985). Participants were instructed to click the left mouse button in their dominant hand as fast as possible and they performed the PVT for 30 min, in line with previous work on the effects of mental fatigue on neuromuscular function (Kowalski & Christie, 2020; Kowalski et al., 2022). When the PVT is performed for 20 min or more, a slowing of reaction time (RT) and reduction in task accuracy occurs indicating mental fatigue (Lim et al., 2010). False starts (FS) where participants click with no stimulus and lapses (RT > 500 ms) were also recorded (Lee et al., 2010; Lim et al., 2010).

### 2.6 Control task

For the control condition, participants watched a 30 min clip of the *Earth* documentary that follows the migration paths of four animal families (Fothergill & Linfield, 2007). This control task has been used previously as an emotionally neutral condition for participants (Kowalski et al., 2022; Pageaux et al., 2013). Sound was transmitted through noise‐cancelling headphones and the documentary was displayed on the same screen utilized for the PVT.

### 2.7 Force

**FIGURE 1:**
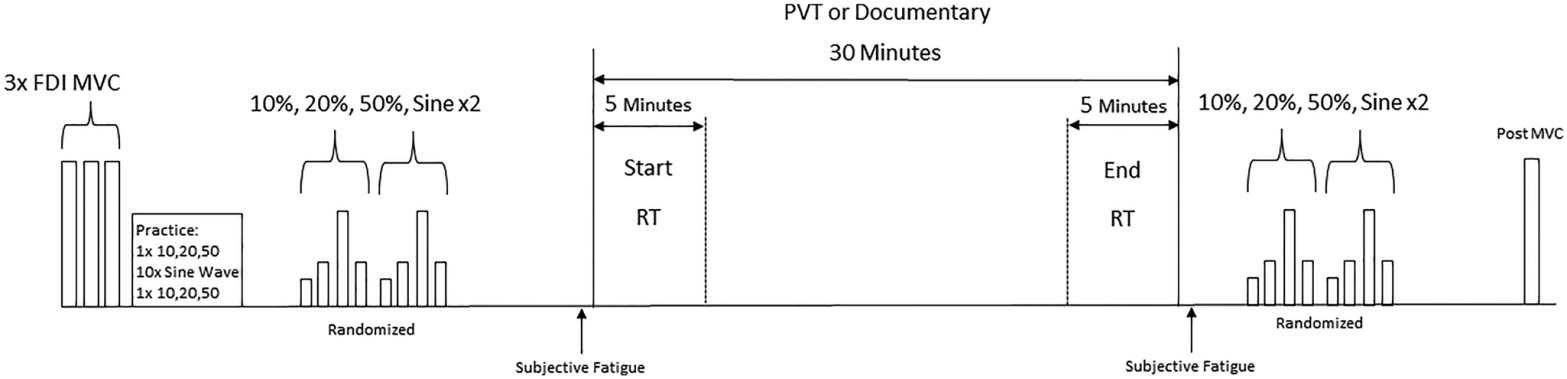
Timeline of experimental protocol. Vertical arrows represent time points and repetitions of subjective fatigue assessments. Horizontal arrows represent time windows.

Participants were seated adjacent to a custom‐built apparatus designed to measure abduction force of the first finger, and their force was displayed in real‐time on a monitor 2 m in front of them. All participants used their non‐dominant FDI with their hand pronated in the device. Participants traced four different force curves: three trapezoidal force curves at 10%, 20% and 50% of their MVC, similar to previous assessments in the TA (Kowalski & Christie, 2020; Kowalski et al., 2022), and a sinusoidal condition at 20 ± 5% MVC at a rate of 0.15 Hz. All force templates traced had a 3 s flat period before the ramp, with a ramp‐up and down‐of 10% MVC/s, and participants performed two of each force tracing before (pre) and after (post) the PVT or documentary. These force tracings were randomized for each participant on each day, and the same randomized order was used for the pre and post conditions on each day. The timeline of the protocol is presented in Figure 1. A custom‐written MATLAB (version 2021a; The MathWorks, Natick, MA, USA) program was used to calculate the root‐mean‐square error (RMSE), mean force and coefficient of variation (CV) of the force. All force outcomes were measured within the middle 3 s of the trapezoidal conditions, and the middle period (∼6.6 s) of the sinusoidal condition. The mean of the two repetitions was used for each pre and post measurement.

### 2.8 EMG signal and motor unit processing

**FIGURE 2:**
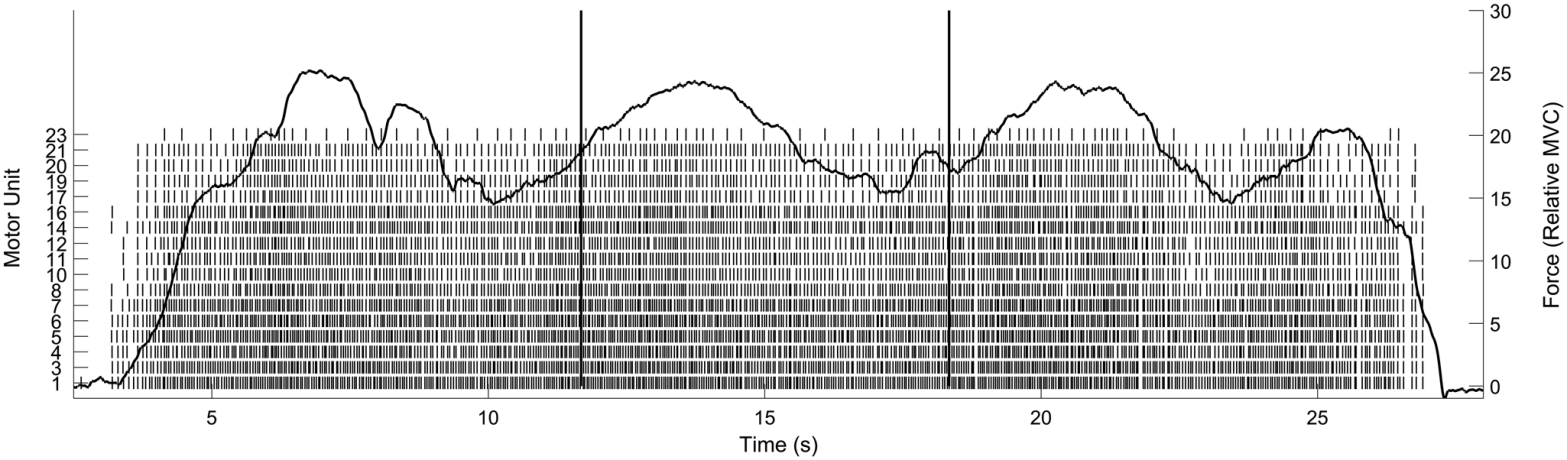
Sample force and motor unit data. Example of a variable force trial for one participant. The identified motor unit firings from the surface decomposition presented underneath a participant's force tracing recording. Motor unit number assigned from the decomposition program is on the left *y*‐axis. The horizontal bars represent the window in which RMSE, CV of force, MUFR and CVISI were calculated.

A wireless four‐pin surface EMG electrode (Galileo wireless EMG, Delsys Inc., Natick, MA, USA) was attached to the posterior of the FDI muscle with a reference electrode attached to the back of the wrist. Surface EMG signals were sampled at a rate of 2222 Hz, band‐pass filtered between 20 and 450 Hz (Delsys Trigno Wireless System, Delsys Inc.) and collected on EMGWorks acquisition software (version 1.2.2, Delsys Inc.). The skin at both the electrode and reference sites was abraded (Nuprep®) and swabbed with alcohol prior to application of the sensor. The sensors were held in place with double‐sided tape, fitted for the electrodes. The EMG signals were decomposed into their motor unit firing trains using the Precision Decomposition III algorithm (Neuromap v1.2; Delsys, Inc.; De Luca et al., 2006; Nawab et al., 2010) and tested for accuracy using the Decompose–Synthesize–Decompose–Compare test (De Luca & Contessa, 2012) using Neuromap software (Delsys Inc.). Briefly, a synthetic signal is generated from the original signal and the decomposition accuracy is compared between the two signals. A complete description of this process can be found elsewhere (Nawab et al., 2010). Motor units below 80% accuracy were excluded from analysis. A threshold of 80% was chosen to perform analysis on a larger set of decomposed motor units similar to previous methodologies (Madarshahian & Latash, 2021; Madarshahian et al., 2021). To reduce the influence of a small number or single instantaneous discharges on the calculation of mean motor unit firing rate, motor unit firings were further excluded if they had fewer than 10 discharges, an inter‐pulse intervals less than 10 ms (doublet discharges), or an inter‐pulse interval greater than 200 ms (Christie & Kamen, 2010; Christie et al., 2009; Inglis & Gabriel, 2020). A custom‐written MATLAB program was used to calculate motor unit firing rates (MUFR) and the variability of the inter‐spike intervals (CVISI). The mean of these values from each of the two trials pre and post for each condition was used. An example of the output of motor unit firings during a variable force contraction is presented in Figure 2.

### 2.9 Statistical analysis

A two‐way (time, sex) repeated‐measures ANOVA was used to determine differences in PVT outcomes (RT, lapses and FS). A three‐way (time, sex, condition) repeated‐measures ANOVA was used to evaluate differences in RMSE, mean force, CV of force, MUFR and CVISI. Student's *t*‐test for independent samples was used to compare participant characteristics between sexes and the survey results from the MFI, PSQI and IPAQ. Statistical analyses were performed with SPSS (Version 28; IBM SPSS Statistics, IBM Corp., Armonk, NY, USA). Outliers within each sex for each force condition were excluded if they were below the first quartile – 1.5 × the interquartile range, or above the third quartile + 1.5 × the interquartile range for any outcome measure. Significance was set at *P* ≤ 0.05, and all data are presented as means ± SD. Effect sizes are presented as partial eta squared (η^2^_p_) and interpreted as small (η^2^_p_ = 0.01), medium (η^2^_p_ = 0.6) and large (η^2^_p_ = 0.14) (Cohen, 1988). Due to technical issues, one male and one female were excluded from the analysis of 10% MVC.

## 3 RESULTS

### 3.1 Participant characteristics

Participant characteristics and survey results are presented in Table 1. Males were taller and heavier than females (*P* < 0.02) with no significant difference in BMI (*P* = 0.9). Males had higher MVC (*P <* 0.001) with no differences between sexes in MFI (*P* = 0.6), PSQI (*P* = 0.8) or IPAQ scores (*P* = 0.2).

**TABLE 1:** Participant characteristics for all 25 participants.

Characteristic	Females	Males
Age (years)	22.5 ± 2.6	24.1 ± 3.1
Height (m)^*^	1.65 ± 0.04	1.81 ± 0.06
Weight (kg)^*^	67.1 ± 15	79.5 ± 8.4
BMI (kg/m^2^)	24.3 ± 5	24.1 ± 2.2
MFI total	61 ± 4.8	59.8 ± 5.6
PSQI	5.3 ± 2.9	4.9 ± 3.4
IPAQ (continuous, METS/week)	4083.1 ± 3091.1	2248.1 ± 2727.0
MVC pre (N)^*^	19 ± 4.6	31 ± 4.8
MVC post (N)^*^	18.1 ± 4.3	28.7 ± 5.1

*Note*: Data are means ± SD.

* Significant difference between sexes (*P* < 0.05). METS = metabolic equivalents.

### 3.2 Mental fatigue

The outcomes from the PVT are presented in Table 2. During the last 5 min of the PVT, participants had a slower reaction time compared to the first 5 min (*P =* 0.001, η^2^ = 0.47). Males had faster RT than females (*P =* 0.02, η^2^ = 0.20), with no significant time by sex interaction (*P =* 0.5). There were no significant main effects of time (*P ≥* 0.08), sex (*P* = 0.2) or time by sex interaction (*P ≥* 0.5) for the number of lapses or false starts. Subjective fatigue reports increased over time on both days (*P ≤* 0.001, η^2^ = 0.41), with a main effect of day (*P =* 0.02, η^2^ = 0.11) in which subjective fatigue reports on the PVT day were higher than the documentary day. There was a main effect of sex on subjective fatigue (*P =* 0.05, η^2^ = 0.08) in which females reported higher feelings of subjective fatigue. There were no significant interactions of time and sex (*P =* 0.3), time and day (*P =* 0.06), day and sex (*P =* 0.7), or time, sex and day (*P =* 0.9) on subjective fatigue.

**TABLE 2:** Psychomotor vigilance task outcomes.

	Start/Pre	End/Post
Subjective fatigue ‐ PVT day*, †		
Females	4.8 ± 1.7	6.2 ± 1.3
Males	3.7 ± 1.3	5.6 ± 1.5
Subjective fatigue ‐ Documentary day*, †		
Females	4 ± 1.3	4.6 ± 1.2
Males	3.4 ± 1.2	4.4 ± 2.2
Reaction time^*^		
Females	269.4 ± 22.1	318.1 ± 48.7
Males	249.8 ± 32.8	286.5 ± 32.2
Lapses		
Females	0.7 ± 0.8	1.1 ± 1.9
Males	1.1 ± 1	2 ± 2.4
False starts		
Females	0.7 ± 0.5	0.3 ± 0.5
Males	0.8 ± 0.5	0.5 ± 0.5

*Note*: Data are means ± SD.

* Indicates significant difference between sexes (*P* < 0.05).

† Indicates significant effect of time (*P* < 0.05).

### 3.3 Force and neuromuscular function

#### 3.3.1 Maximal voluntary contraction

For each force level, any participants with outliers for RMSE, CV or mean force within each sex were excluded. For MVC, one female and one male were excluded as outliers. There was a main effect of time on MVC (*P <* 0.001, η^2^ = 0.27) where MVC was reduced over time. There was also a significant effect of sex (*P <* 0.001, η^2^ = 0.64) where males were higher than females at both time points. There was no significant effect of day (*P* = 0.9), nor any significant interactions (*P* ≥ 0.07).

#### 3.3.2 Ten percent contraction intensity

**FIGURE 3:**
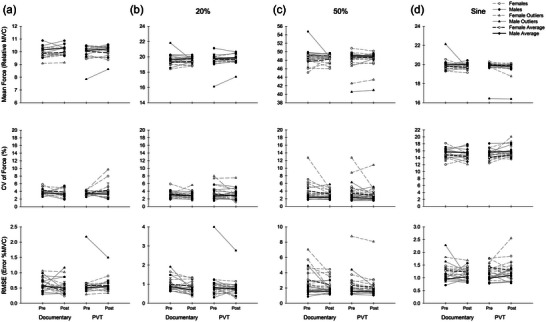
Force outcomes. Individual data are presented with grey lines and mean data presented in black. Statistical outliers (not included in analyses) are presented as triangles. (a) 10% MVC. There were no significant changes over time, nor any effects of sex or day. There were no significant effects interactions on force outcomes at 10% MVC. (b) 20% MVC. RMSE was significantly reduced over time (*P* = 0.02) in both sexes. (c) 50% MVC. In both sexes, CV of force was reduced over time (*P* = 0.03). Males had higher mean force (*P* = 0.005), lower CV of force (*P* < 0.001) and less error (*P* < 0.001) than females. (d) Sine wave. Males had less error than females (*P* < 0.001), but there was an improvement (reduction) in error in females (*P* < 0.05). Males also had higher CV of force (*P* = 0.005).

Force outcomes for 10% MVC are presented in Figure 3. For the 10% MVC contractions, four females and three males were excluded from the analysis as outliers and one female and one male were excluded due to technical issues with collection, and therefore the analysis was performed on seven females and seven males. Mean and CV of force had no significant main effect of time (*P ≥* 0.3, η^2^ ≤ 0.05), sex (*P ≥* 0.06, η^2^ ≤ 0.14) or day (*P ≥* 0.8, η^2^ ≤ 0.004). Although the sex effect was not significant for CV of force, there was a large effect size (η^2^ = 0.14), as the mean CV was greater in males. There were no significant interactions for mean (*P* ≥ 0.2, η^2^ ≤ 0.08) or CV of force (*P* ≥ 0.3, η^2^ ≤ 0.05). The RMSE of force at 10% had no main effects of time (*P* = 0.8, η^2^ = 0.002), sex (*P* = 0.8, η^2^ = 0.003) or day (*P* = 0.9, η^2^ ≤ 0.001), with no significant interactions (*P* ≥ 0.2, η^2^ ≤ 0.06).

**FIGURE 4:**
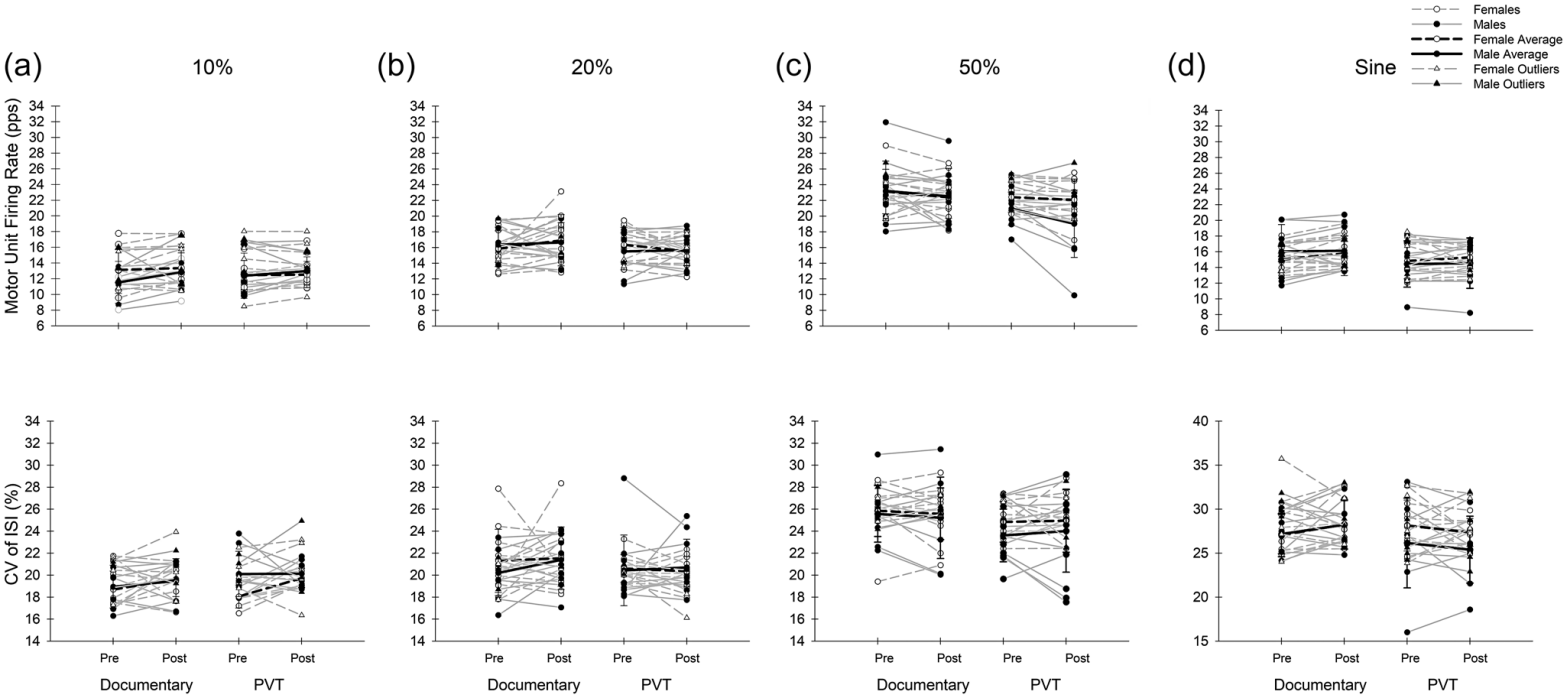
Motor unit firing behaviour. Individual data are presented with grey lines and mean data presented in black. Statistical outliers (not included in analyses) are presented as triangles. (a) 10% MVC. Motor unit firing rate was higher at post compared to pre (*P* = 0.04) in both sexes. There were no significant changes over time, nor any effects of sex or day. There were no significant interactions on CVISI at 10% MVC. (b) 20% MVC. No significant changes over time, nor any interactions for MUFR at 20% MVC. (c) 50% MVC. Motor unit firing rate at 50% MVC was lower at post compared to pre (*P* = 0.02). There were no significant effects of sex or day. There were no significant interactions on CVISI at 50% MVC. (d) Sine wave. There were no significant changes over time, nor effects of sex or day. No significant interactions were observed for MUFR or CVISI during the sine wave contraction.

On the documentary day, 133 motor units were identified before the intervention and 143 after. On the PVT day, 143 motor units were identified before the intervention and 139 after. Motor unit firing behaviour outcomes for 10% MVC are presented in Figure 4. Motor unit firing rate at 10% significantly increased over time (*P* = 0.04, η^2^ ≥ 0.16), with no significant effect of sex (*P* = 0.06, η^2^ = 0.09) or day (*P* = 0.9, η^2^ = 0.001). There were no significant interactions for MUFR (*P* ≥ 0.1, η^2^ ≤ 0.09). The CVISI had no significant effects of time (*P* = 0.09, η^2^ = 0.11), sex (*P* = 0.08, η^2^ ≥ 0.12) or day (*P* = 0.2, η^2^ ≥ 0.06). There were also no significant interactions for CVISI (*P* ≥ 0.09, η^2^ ≤ 0.11).

#### 3.3.3 Twenty percent contraction intensity

Force outcomes for 20% MVC are presented in Figure 3. During the 20% MVC contractions, two females and two males were excluded from the analysis as outliers, therefore the analysis was performed on 11 females and 10 males. There was no significant change over time in mean (*P* = 0.2, η^2^ = 0.05) or CV of force (*P* = 0.09, η^2^ = 0.07). Males had higher mean force compared to females (*P* = 0.04, η^2^ = 0.1), but there was no significant difference in CV of force (*P* = 0.6, η^2^ ≥ 0.008). There were no significant interactions for mean force (*P* ≥ 0.4, η^2^ ≤ 0.08) or CV of force (*P* ≥ 0.2, η^2^ ≤ 0.05). The RMSE of force at 20% was reduced over time (*P* = 0.02, η^2^ = 0.13), with no significant main effects of sex (*P* = 0.2, η^2^ = 0.03) or day (*P* = 0.2, η^2^ = 0.08) and no significant interactions (*P* ≥ 0.3, η^2^ ≤ 0.03).

On the documentary day, 346 motor units were identified before the intervention and 370 after. On the PVT day, 342 motor units were identified before the intervention and 337 after. Motor unit firing behaviour outcomes for 20% MVC are presented in Figure 4. Motor unit firing rate at 20% had no significant main effects of time (*P* ≥ 0.6, η^2^ = 0.006), sex (*P* = 0.8, η^2^ = 0.001) or day (*P* = 0.3, η^2^ = 0.03). There were no significant interactions for MUFR (*P* ≥ 0.2, η^2^ ≤ 0.04). The CVISI at 20% had no significant effects of time (*P* = 0.4, η^2^ = 0.01), sex (*P* = 0.7, η^2^ = 0.005) or day (*P* = 0.3, η^2^ = 0.02). There were also no significant interactions for CVISI (*P* ≥ 0.4, η^2^ ≥ 0.02).

#### 3.3.4 Fifty percent contraction intensity

Force outcomes for 50% MVC are presented in Figure 3. During the 50% MVC contractions, one female and three males were excluded as outliers. Therefore, the analysis was performed on 12 females and nine males. Over time, mean force did not significantly change (*P* = 0.6, η^2^ = 0.009), but CV of force was significantly reduced (*P* = 0.03, η^2^ = 0.11). There was a significant effect of sex for both mean force (*P* = 0.005, η^2^ = 0.19) and CV of force (*P* < 0.001, η^2^ = 0.35) in which males had a higher mean force and were less variable than females. There was no significant effect of day on mean force (*P* = 0.2, η^2^ = 0.05) or CV of force (*P* = 0.2, η^2^ = 0.04). There were no significant interactions for mean force (*P* ≥ 0.1, η^2^ ≤ 0.07) or CV of force (*P* ≥ 0.2, η^2^ ≤ 0.05). The RMSE of force at 50% did not significantly change over time (*P* = 0.06, η^2^ = 0.09). Compared to females, males had significantly lower RMSE values (*P* ≤ 0.001, η^2^ = 0.34). There was no significant effect of day on RMSE (*P =* 0.1, η^2^ = 0.05). There were also no significant interactions for RMSE (*P* ≥ 0.1, η^2^ ≤ 0.06).

On the documentary day, 647 motor units were identified before the intervention and 633 after. On the PVT day, 609 motor units were identified before the intervention and 515 after. Motor unit firing behaviour outcomes for 50% MVC are presented in Figure 4. Motor unit firing rate at 50% significantly decreased over time (*P* = 0.01, η^2^ = 0.15), but there were no significant effects of sex (*P* = 0.2, η^2^ = 0.04) or day (*P* = 0.6, η^2^ = 0.09). There were no significant interactions for MUFR (*P* ≥ 0.2, η^2^ ≤ 0.05). The were no significant effects of time (*P* = 0.9, η^2^ < 0.001), sex (*P* = 0.4, η^2^ = 0.02) or day (*P* = 0.1, η^2^ = 0.05) on CVISI. There were also no significant interactions for CVISI (*P* ≥ 0.3, η^2^ ≤ 0.2) during the 50% contractions.

#### 3.3.5 Sinusoidal contraction

Force outcomes for the sinusoidal contractions are presented in Figure 3. During the sinusoidal contractions, five females and four males were excluded as outliers. Therefore, the analysis was performed on eight females and eight males. Neither mean force (*P* = 0.2, η^2^ = 0.07) nor CV of force (*P* = 0.7, η^2^ = 0.005) significantly changed over time. Males had more variable force than females (*P* = 0.005, η^2^ = 0.24), but mean force was not significantly different between sexes (*P* = 0.3, η^2^ = 0.04). There was no significant effect of day on mean force (*P* = 0.9, η^2^ = 0.001) or CV of force (*P* = 0.9, η^2^ < 0.001). There were no significant interactions for mean force (*P* ≥ 0.1, η^2^ ≤ 0.07) or CV (*P* ≥ 0.2, η^2^ ≤ 0.05) of force in the sinusoidal contraction. The RMSE did not significantly change over time (*P* = 0.9, η^2^ = 0.001), but there was a significant effect of sex (*P* < 0.001, η^2^ = 0.46) in which males were more variable than females. There was no effect of day on RMSE (*P* = 0.2, η^2^ = 0.05). There was a significant interaction of time and sex on the RMSE during the sinusoidal contraction (*P* = 0.05, η^2^ = 0.13) where males had increased error over time and females reduced their error over time. There were no additional significant interactions for RMSE (*P* ≥ 0.1, η^2^ ≤ 0.06).

On the documentary day, 437 motor units were identified before the intervention and 506 after. On the PVT day, 424 motor units were identified before the intervention and 410 after. Motor unit firing behaviour outcomes for the sinusoidal contractions are presented in Figure 4. Motor unit firing rate during the sinusoidal contraction had no effect of time (*P* = 0.2, η^2^ = 0.07), sex (*P* = 0.9, η^2^ < 0.001) or day (*P* = 0.3, η^2^ = 0.04). There were no significant interactions for MUFR (*P* ≥ 0.3, η^2^ ≤ 0.05). There was no significant effect of time (*P* = 0.7, η^2^ = 0.005), sex (*P* = 0.3, η^2^ = 0.03) or day (*P* = 0.3, η^2^ = 0.03) on CVISI. There were no significant interactions for CVISI (*P* ≥ 0.06, η^2^ ≤ 0.11) during the sinusoidal contractions.

## 4 DISCUSSION

In this study we sought to further investigate potential sex‐related differences in the impact of mental fatigue on neuromuscular function in the FDI. The PVT successfully induced mental fatigue, as indicated by a slowed reaction time and higher reported subjective fatigue. In this study, we did not observe any significant changes to force or motor unit firing behaviour in the FDI that was specific to mental fatigue. Motor unit firing rates increased over time on both days in the 10% condition and decreased in the 50% conditions with no change to the mean force, and a decrease in the force variability. However, these changes were not specific to the mental fatigue condition. We did observe that females reduced their error in tracing a sinusoid force pattern, whereas males increased their error.

### 4.1 Mental fatigue

Our 30‐min PVT task seems to have successfully induced mental fatigue, as indicated by a significant slowing of RT. On both days, participants reported an increase in subjective fatigue, with higher reported values observed on the PVT day at both time points. The large change in RT is similar to previous assessments of mental fatigue using the PVT (Kowalski & Christie, 2020; Kowalski et al., 2022; Morris & Christie, 2020). Females reported greater subjective fatigue than males at both time points irrespective of mental fatigue, supporting previous findings that females report greater subjective fatigue (Engberg et al., 2017). Our observed increase in subjective fatigue over time was not specific to mental fatigue, although this did trend towards significance on the PVT day. Previous studies observed increases of 14–19.5% in subjective fatigue reports after sustained attention tasks (Kowalski & Christie, 2020; Pageaux et al., 2015), which is slightly lower than our changes of 12–33% change in subjective fatigue. These larger changes could have been driven by our population's scores on the PSQI, where females reported an average of 5.3 and males of 4.9, where 5 and higher is considered poor sleep. Our average MFI scores of 61 for females and 59.8 for males were similar to previous assessments of neuromuscular function and mental fatigue, ranging from 59.3 to 60 (Kowalski & Christie, 2020; Morris & Christie, 2020).

### 4.2 Maximal force

The sex difference in MVC in our participants was large, similar to previous assessments of MVC in the FDI (Parra et al., 2020). Maximal voluntary contraction was also reduced over time by an average of 5%, which, while significant, is a much lower change compared to the fatigue observed in isometrically fatiguing studies on the FDI (McManus et al., 2015). There was also no combination of any other changes in mean or variability of force over time to indicate that fatigue was an influence in performance. Other studies performed on dorsiflexion and knee extension found no difference in MVC over time, with similar differences in MVC force between sexes where males were stronger (Kowalski & Christie, 2020; Kowalski et al., 2022; Pageaux et al., 2013, 2015). However, in the hand, significant reductions were found in the present study and during an isometric handgrip task (Bray et al., 2012) in contrast to others showing no change (Bray et al., 2008; Mehta & Parasuraman, 2014).

The methodologies of these studies could be influencing the variability of the responses to mental fatigue. For example, maximal handgrip tasks require more musculature than first finger abduction or ankle dorsiflexion. In previous studies with similar protocols in the TA, we did not find a reduction in MVC over time following a similar mental fatigue and control task (Kowalski & Christie, 2020; Kowalski et al., 2022). The contrasting finding of a significant reduction in MVC in the current study may be related to differences in the function of the two muscles. For example, the FDI has fewer motor units, a lower innervation ratio and a smaller recruitment range compared with the TA (De Luca & Hostage, 2010; Duchateau & Enoka, 2022; van Cutsem et al., 1997). Therefore, during the submaximal contractions of our protocol, the FDI should be recruiting relatively more motor units than the TA during similar contractions. It is possible that these physiological differences create a different perception of effort throughout the task. Although our subjective fatigue ratings were not different from previous reports (Kowalski & Christie, 2020; Kowalski et al., 2022), we did not assess perception of effort. It is also possible other factors not assessed in this study contribute to changes in MVC force following a mentally fatiguing task. Further work is necessary in this area to fully understand potential differences in the neuromuscular response to mental fatigue under different task conditions and in different muscles.

### 4.3 Submaximal force

During the submaximal contractions, we did not observe any changes to RMSE, CVISI or mean force that were unique to the mentally fatiguing condition. This is consistent with previous findings on force steadiness in the FDI following mental fatigue (Budini et al., 2022) and handgrip steadiness (Shortz & Mehta, 2017). Males in our study had higher mean force than females at 20% MVC, and less error and RMSE with higher mean force during the 50% MVC trapezoidal contractions. This is in line with previous sex‐based comparisons with lower CV of force and RMSE in males (Inglis & Gabriel, 2021; Jakobi et al., 2018). A reduction in CV of force and RMSE over time occurred at 50% and 20%, respectively, although these were not unique to mental fatigue. This reduction was not observed in the sinusoidal contraction. The practice session included a focus on the sinusoidal contraction, and less on the static contractions which could have led to this change over time.

At 10% MVC, there were no significant changes to the force output, but there was a trend for male CV of relative force to be higher than females. Despite any changes to force outcomes, there was a significant increase in MUFR over time. The average firing rate at the pre time point was about 12.4 pulses per second, and at the post time point was about 13 pulses per second. The effect size of this change was large, and the increase in MUFR with a lack of differences in mean, CV and RMSE of force is in contrast to a previous assessment of MUFR in the tibialis anterior in a similar assessment of mental fatigue, where a decline in average force was found with no change to MUFR (Kowalski et al., 2022). A previous study on the TA also suggested that mental fatigue could have changed recruitment or synergistic muscle activity (Kowalski et al., 2022). However, a lack of significant contributors of synergistic muscle activity in the FDI indicates that this is likely not a factor in this study. These contrasting outcomes indicate support for these outcomes being different across muscles.

At 20% MVC, we observed a reduction in RMSE over time in both sexes, and males traced the templates with a higher relative mean force than females. This reduction in RMSE was not accompanied by other changes in force performance. We did not observe any changes to MUFR on the PVT or documentary day, in contrast to previous studies in the tibialis anterior, where a reduction in MUFR was observed in response to the same conditions (Kowalski & Christie, 2020; Kowalski et al., 2022). The different responses in MUFR to mentally fatiguing conditions between studies could potentially be attributed to the muscles examined.

The 50% MVC condition had significant changes over time to both the CV of force and the MUFR, as well as sex differences in RMSE, mean and CV of force. Mean force output during the 50% condition was higher and less variable in males than females. However, both sexes demonstrated a significant reduction in CV of force over time. There was a trend for RMSE at 50% to reduce over time in both sexes, but it failed to reach significance. This decline in MUFR at 50% MVC is in‐line with one previous assessment of MUFR in the tibialis anterior (Kowalski & Christie, 2020), but not when compared to another assessment which included a documentary condition as well (Kowalski et al., 2022).

A novel observation in the present study was in the performance of the sinusoidal force tracing. Similar to the complex sinusoidal wave utilized in the FDI previously (Knight & Kamen, 2007), a flat rate of 0.15 Hz was utilized. Males had less error in force in performing the task yet were more variable during their performance. Interestingly, over time males also showed an increase in their RMSE, while females reduced their error. Our females also reported higher levels of subjective fatigue and scored over 5 on the PSQI on average, which could have potentially had an influence on their performance. The differences in RMSE performance could be related to motor unit recruitment, as it has been suggested, based on motor unit action potential size, that in the FDI males have larger motor units than females (Parra et al., 2020), suggesting females may rely more on new recruitment to increase force. Our variable force contraction varied around this percentage, and potentially this sex difference in size of motor units during the variable force condition could have contributed to the sex‐specific improvement. In another study using a force‐tracing task with a complex sinusoidal wave, a group of female participants increased their performance through a reduction in RMSE by 52.8% after 15 trials, which was accompanied by improvements in modulation frequencies of discharge rate specific to the task (Knight & Kamen, 2004). A lack of sex differences in MUFR and the CVISI with an improvement in RMSE in female performance during the variable force condition, observed in the current study could indicate sex differences in the modulation of firing rates during variable force‐matching tasks at 0.15 Hz.

### 4.4 Limitations

Most of our PVT outcomes were indicative that the PVT successfully induced mental fatigue. However, we observed an increase in subjective fatigue reported on both days, contrary to a similar protocol using the same control stimulus (Kowalski et al., 2022). Our participants also did not experience any significant changes in false starts or lapses, potentially indicating different levels of mental fatigue in response to the PVT in our participants compared to the previous study. The PVT is a simple reaction time task, and as such maybe the length of the task combined with simpler nature compared with other tasks like the Stroop was not enough to induce levels of mental fatigue required to observe neuromuscular changes. The length of the protocol following the PVT trial could also have exceeded the duration of the effects of mental fatigue; however, our protocol was completed within 10–12 min, before the suggested 15–45 min range of suggested recovery time from mental fatigue (Magnuson et al., 2021). In terms of neuromuscular outcomes following mental fatigue, it is possible the recovery duration is different across different muscles. Mental fatigue recovery is an area that should be explored further. Furthermore, the varying numbers of outliers across the conditions could have influenced the outcomes. However, a separate analysis including only participants with complete data sets did not change the outcomes of the study.

## 5 CONCLUSION

Overall, our findings indicate that there is a lack of significant change to neuromuscular function in response to a mentally fatiguing task, as there was no unique change compared to a control condition. It is therefore unlikely that neuromuscular factors explain previously reported changes in performance following mental fatigue (Pageaux & Lepers, 2018; van Cutsem et al., 2017). Although there is a lack of significant change to motor unit firing behaviour in response to mental fatigue, there is evidence to suggest that increased feelings of subjective fatigue can cause different adaptations to neuromuscular function according to the muscle. The changes we observed were not unique to the mental fatigue condition. Changes over time were observed during both a mentally fatiguing task and a mentally neutral documentary. Females in our study improved in their performance of tracing a sinusoid force pattern while males had a reduction in performance over time. Sex differences were observed in force tracing performance of a variable force task, and these results indicate that more sex‐specific comparisons of performance during variable force contractions should be made.

## AUTHOR CONTRIBUTIONS

Michael J. Marsala contributed to study conception and design, data collection, analysis, and interpretation, and participated in writing and editing of the mansucript. Anita D. Christie contributed to study conception and design, data interpret, and writing and editing of the manuscript. Both authors have approved the final version of the manuscript and agree to be accountable for all aspects of the work in ensuring that questions related to the accuracy or integrity of any part of the work are appropriately investigated and resolved. All persons designated as authors qualify for authorship, and all those who qualify for authorship are listed.

## CONFLICT OF INTEREST

The authors have no conflicts of interest to declare.

## Data Availability

All data are included in the manuscript figures.
